# Green Synthesis of Encapsulated Copper Nanoparticles Using a Hydroalcoholic Extract of *Moringa oleifera* Leaves and Assessment of Their Antioxidant and Antimicrobial Activities

**DOI:** 10.3390/molecules25030555

**Published:** 2020-01-28

**Authors:** Prince Edwin Das, Imad A. Abu-Yousef, Amin F. Majdalawieh, Srinivasan Narasimhan, Palmiro Poltronieri

**Affiliations:** 1 Asthagiri Herbal Research Foundation, 162A, Perungudi Industrial Estate, Perungudi, Chennai 600096, India; prince.ahrf@gmail.com; 2 Department of Biology, Chemistry and Environmental Sciences, American University of Sharjah, P.O. Box 26666 Sharjah, UAE; amajdalawieh@aus.edu; 3 Institute of Sciences of Food Productions, CNR-ISPA, 73100 Lecce, Italy

**Keywords:** *Moringa oleifera*, copper nanoparticles, polyphenolics, anti-bacterial, anti-fungal, antioxidant

## Abstract

The synthesis of metal nanoparticles using plant extracts is a very promising method in green synthesis. The medicinal value of *Moringa oleifera* leaves and the antimicrobial activity of metallic copper were combined in the present study to synthesize copper nanoparticles having a desirable added-value inorganic material. The use of a hydroalcoholic extract of *M. oleifera* leaves for the green synthesis of copper nanoparticles is an attractive method as it leads to the production of harmless chemicals and reduces waste. The total phenolic content in the *M. oleifera* leaves extract was 23.0 ± 0.3 mg gallic acid equivalent/g of dried *M. oleifera* leaves powder. The *M. oleifera* leaves extract was treated with a copper sulphate solution. A color change from brown to black indicates the formation of copper nanoparticles. Characterization of the synthesized copper nanoparticles was performed using ultraviolet-visible light (UV-Vis) spectrophotometry, Fourier-transform infrared (FTIR) spectrometry, high-resolution transmission electron microscopy (HRTEM), scanning electron microscopy (SEM), and X-ray diffraction (XRD). The synthesized copper nanoparticles have an amorphous nature and particle size of 35.8-49.2 nm. We demonstrate that the *M. oleifera* leaves extract and the synthesized copper nanoparticles display considerable antioxidant activity. Moreover, the *M. oleifera* leaves extract and the synthesized copper nanoparticles exert considerable anti-bacterial activity against *Escherichia coli*, *Klebsiella pneumoniae*, *Staphylococcus aureus*, and *Enterococcus faecalis* (MIC values for the extract: 500, 250, 250, and 250 µg/mL; MIC values for the copper nanoparticles: 500, 500, 500, and 250 µg/mL, respectively). Similarly, the *M. oleifera* leaves extract and the synthesized copper nanoparticles exert relatively stronger anti-fungal activity against *Aspergillus niger*, *Aspergillus flavus*, *Candida albicans*, and *Candida glabrata* (MIC values for the extract: 62.5, 62.5, 125, and 250 µg/mL; MIC values for the copper nanoparticles: 125, 125, 62.5, and 31.2 µg/mL, respectively). Our study reveals that the green synthesis of copper nanoparticles using a hydroalcoholic extract of *M. oleifera* leaves was successful. In addition, the synthesized copper nanoparticles can be potentially employed in the treatment of various microbial infections due to their reported antioxidant, anti-bacterial, and anti-fungal activities.

## 1. Introduction

*Moringa oleifera* (family Moringaceae) is known as the “tree of life”. For thousands of years, it has been widely cultivated for its industrial and medicinal value. It is cultivated for its leaves and fruits, which are commonly used for cooking. Almost all parts of the plant have been utilized in home remedies and traditional medicine [1]. *M. oleifera* leaves are edible and their vitamin and amino acid contents make them a well-balanced addition to a diet [2]. The phenolic compounds present in *M. oleifera* leaves possess antioxidant properties and they are used in various medical applications [3]. It has been envisaged that plant compounds and their derivatives may be applied in several fields and industries such as textiles, fabrics, polymers for food and non-food applications, biomaterials for oral hygiene and prevention of dental caries, prevention of biofilm formation, and wrapping foil polymers for food packaging [4,5,6,7,8,9]. New materials synthesized based on the antimicrobial properties of silver nanoparticles and other nanoparticle-related technologies have been produced and studied [10,11,12,13,14,15].

Previous studies reported the successful use of active components of plant extracts (e.g., proteins, flavonoids and carboxylic groups of arabinose and galactose, reducing sugars, tannins, aliphatic amines, aliphatic alkenes of alkaloids, polysaccharides, aromatic amines, sec-alcohols, water-soluble heterocyclic components and saponins) in the synthesis of silver nanoparticles [16]. Typically, plant extracts possess intrinsic biological activities, which may further manifest in the biological activities of silver nanoparticles as a result of combining the two materials. Therefore, plant extracts can potentially be developed into novel nanomaterials with diverse biological activities [17]. Gold and silver nanoparticles were synthesized using leaf extracts of *Erythrina suberosa (Roxb.)*, *Paederia foetida*, *Acalypha indica*, *Cassia auriculata*, *Sorbus aucuparia*, and *Azadirachta indica*, and their antimicrobial efficacy was evaluated [18,19,20,21,22,23]. Moreover, other studies evaluated the in vitro antioxidant and antimicrobial activities of silver nanoparticles that were synthesized using various plant extracts [24,25,26,27,28]. In many cultures, people used to, and some still do, drink water stored in copper vessels. Such water has a copper content of 177 ± 16 ppb, which is well within the permissible limits according to the World Health Organization (WHO) [29]. Storing water in copper vessels purifies water by killing some species and strains of bacteria like *Escherichia coli*, as metallic copper surfaces rapidly and efficiently destroy bacteria through a contact-killing mode [30].

Our study attempts to bring together the properties of *M. oleifera* leaves’ phytochemical constituents and the antimicrobial activity of copper. We aimed at the green synthesis and characterization of copper nanoparticles using a hydroalcoholic extract of *M. oleifera* leaves. Using different species of bacteria and fungi, we evaluated the potential anti-bacterial and anti-fungal activities of the *M. oleifera* leaf extract and the synthesized copper nanoparticles. The potential advantages of copper nanoparticles synthesized using plant extracts are their applications related to medicinal therapies, dental remedies, water treatment, and lotions. The anti-bacterial properties of copper nanoparticles against *Bacillus subtilis* and *Staphylococcus aureus* were reported [31]. The following few salient points support our attempt toward the green synthesis of copper nanoparticles:

Copper nanoparticles synthesized using plant extracts had prospective applications as antimicrobial agents over silver nanoparticles due to the low cost of copper compared to silver.

A phytochemical-encapsulated copper nanomaterial has an advantageous feature for direct topical applications.

The process of synthesis adopted in this study is intensively clean, cost-effective, and efficient [32].

## 2. Results and Discussion

### 2.1. Phytochemical Analysis of the M. oleifera Leaves Extract

The qualitative evaluation of different chemical constituents in the *M. oleifera* leaves extract was performed using the test methods indicated in Table 1 [33]. The presence is indicated with (+).

**Figure 1:**
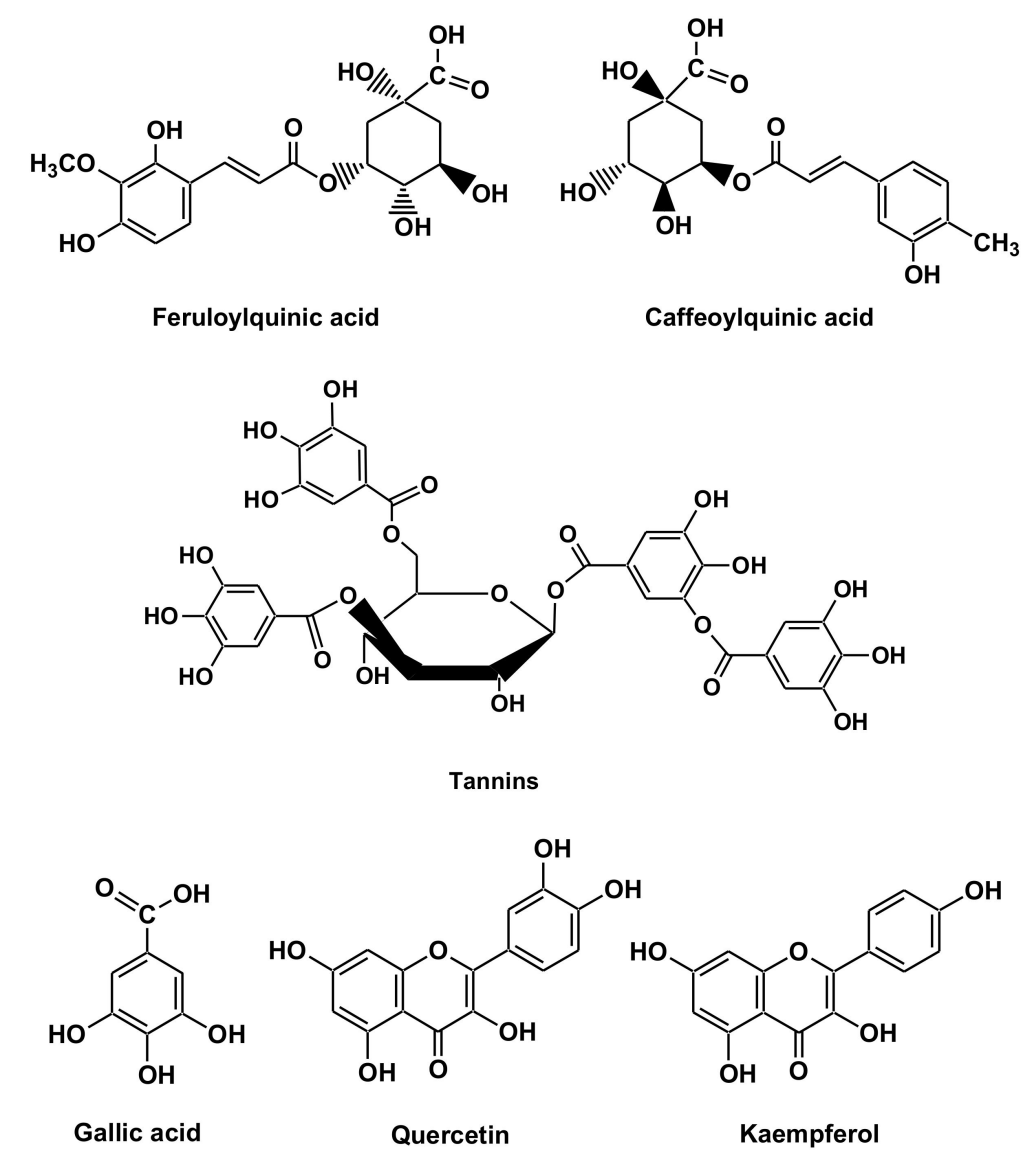
Chemical structures of major phytochemicals present in *M. oleifera* leaves extract.

The protein content in the *M. oleifera* leaves extract was estimated to be 0.1% of the dry leaves powder. The total phenolic content in *M. oleifera* leaves extract was 23% of the dry leaves powder. An earlier study demonstrated that alanine, tyrosine, lysine, and threonine are among the major amino acids present in the *M. oleifera* leaves extract [34]. The concoction of *M. oleifera* leaves extract contains isomers of caffeoylquinic acid, isomers of feruloylquinic acid, tannins, gallic acid, and several flavonoids like quercetin, kaempferol and their glycoside derivatives [35]. The chemical structures of these compounds are shown in Figure 1. Collectively, these findings suggest that the *M. oleifera* leaves extract could serve as a nutritional supplement and a possible stabilizing agent for the formed copper nanoparticles.

**Scheme 1:**
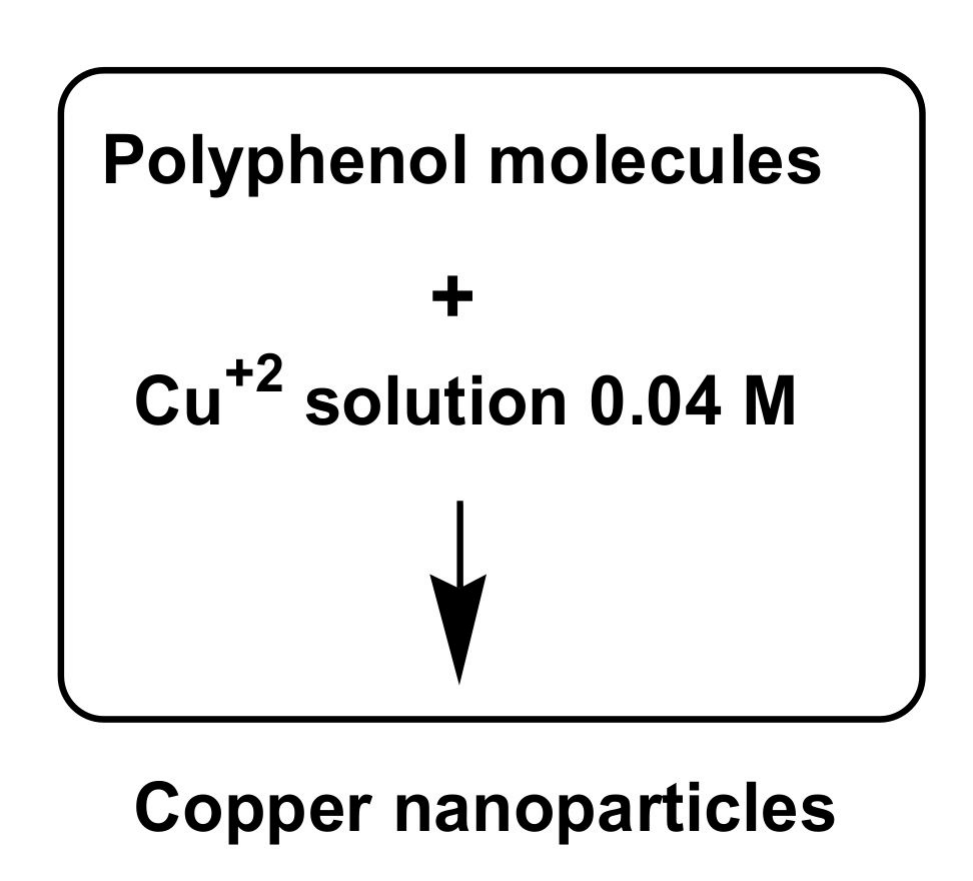
Schematic representation of the synthesis of copper nanoparticles.

Based on the total phenolic content estimation of the *M. oleifera* leaves extract before and after the reaction (Table 2), Scheme 1 was proposed. The total phenolic content of 60 mg gallic acid equivalent from 10 g of dried *M. oleifera* leaves powder was used in the synthesis of 250 mg of copper nanoparticles from 0.04 M of copper (II) ion solution. The other reducing and binding chemical entities present in the concoction aid in the formation and stabilization of the synthesized copper nanoparticles by encapsulating the nanomaterial. Comparing the total polyphenol content before and after the synthesis, there was a reduction in total polyphenol content. This substantiates the deduction that some polyphenols are involved in the bio-reduction and subsequent stabilization of the nanoparticles by encapsulation.

### 2.2. Characterization

#### 2.2.1. Size and Morphology of the Synthesized Copper Nanoparticles

**Figure 2:**
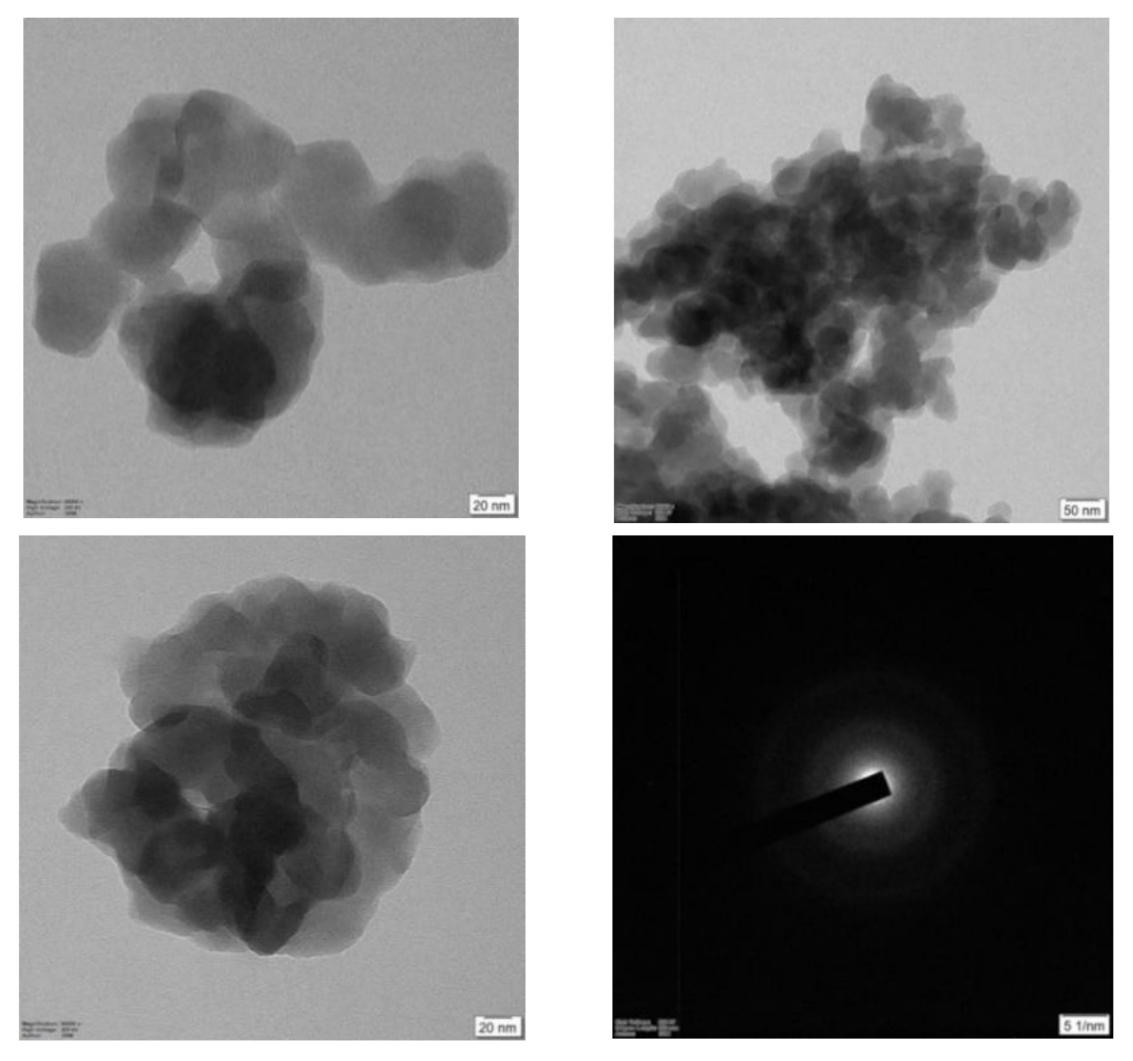
Transmission electron microscopy (TEM) images of the synthesized copper nanoparticles.

**Figure 3:**
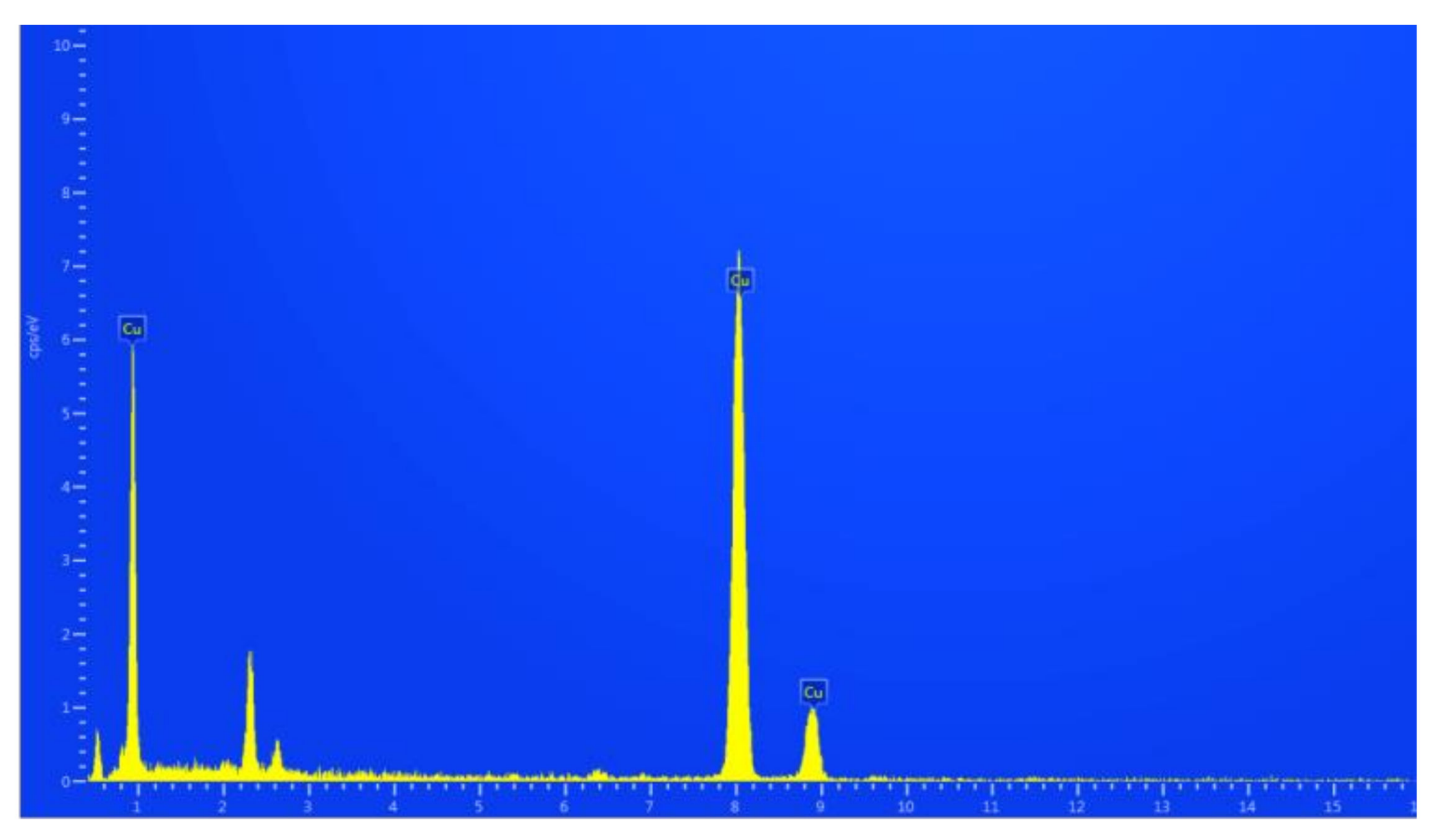
Energy dispersive X-ray spectroscopy (EDS) spectrum with peaks corresponding to copper.

The high-resolution transmission electron microscopy (HRTEM) image of synthesized copper nanoparticles was captured using a JEOL-TEM 2100 plus electron microscope (Figure 2). EDS (energy dispersive X-ray spectroscopy) studies indicate only the presence of elements in the sample (Figure 3).

**Figure 4:**
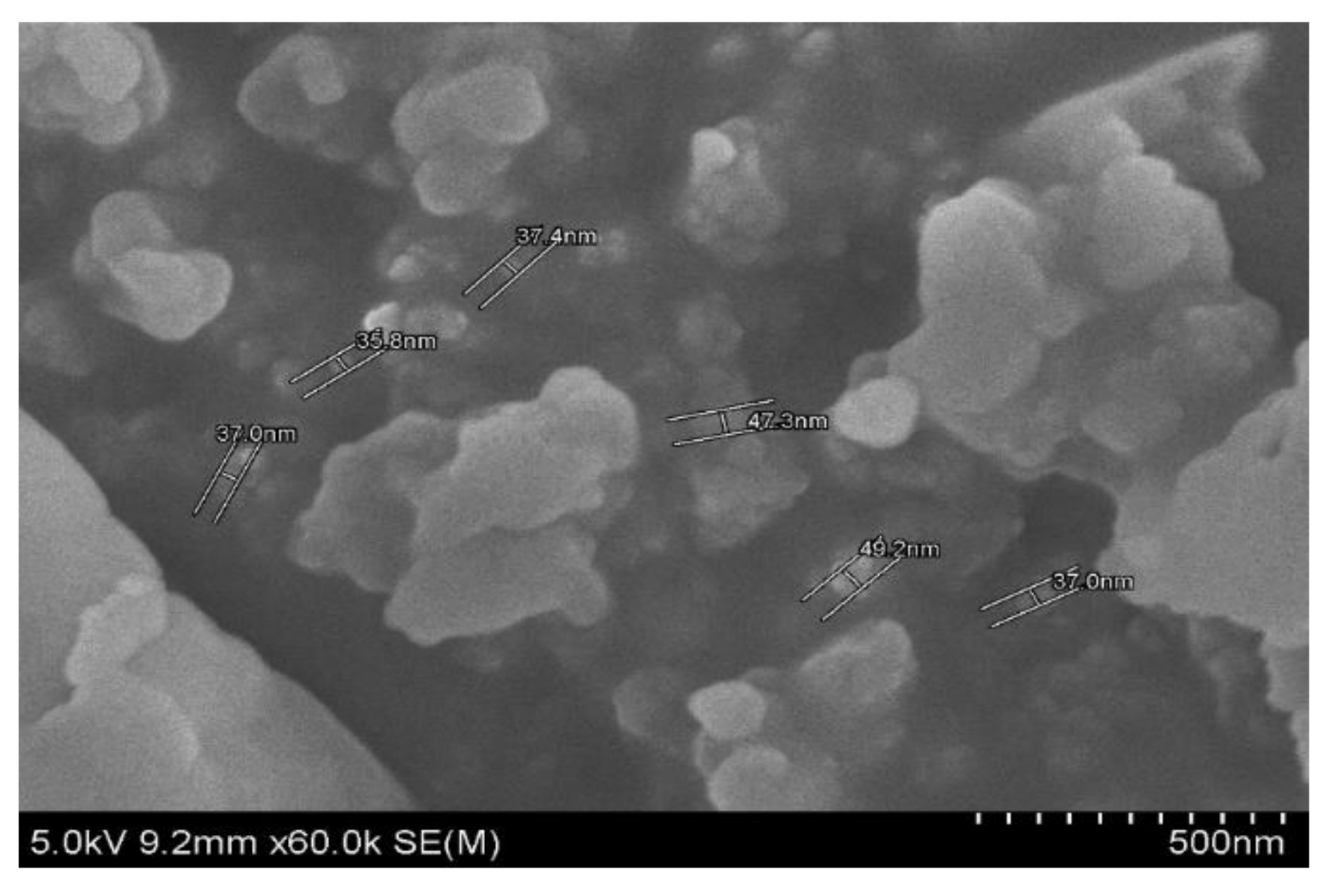
Scanning electron microscopy (SEM) image of the synthesized copper nanoparticles.

The synthesized copper nanoparticles were obtained in ethanol to give a colloidal solution. The synthesized copper nanoparticles are amorphous in nature and they agglomerate upon storage. EDS analysis confirmed the presence of the elemental copper nanoparticles with Kα line at 8.04 and Lα line at 0.92 (X-ray energies for copper). The unlabeled peaks correspond to sulphur (Kα line at 2.3) and oxygen (Kα line at 0.52). These two elements can appear from sulphur-containing phytochemicals that had been encapsulated into the nanomaterial. The absence of other elemental peaks reflects the purity of the sample. The scanning electron microscopy (SEM) images (Figure 4) indicate that the size of the synthesized copper particles is 35.8–49.2 nm. The macromolecules that encapsulated the copper nanoparticles promote the agglomeration of the nanomaterial in layers. So, the smallest grains give the best chance to capture the size of the nanoparticle, as the effect of agglomeration will be negligible.

**Figure 5:**
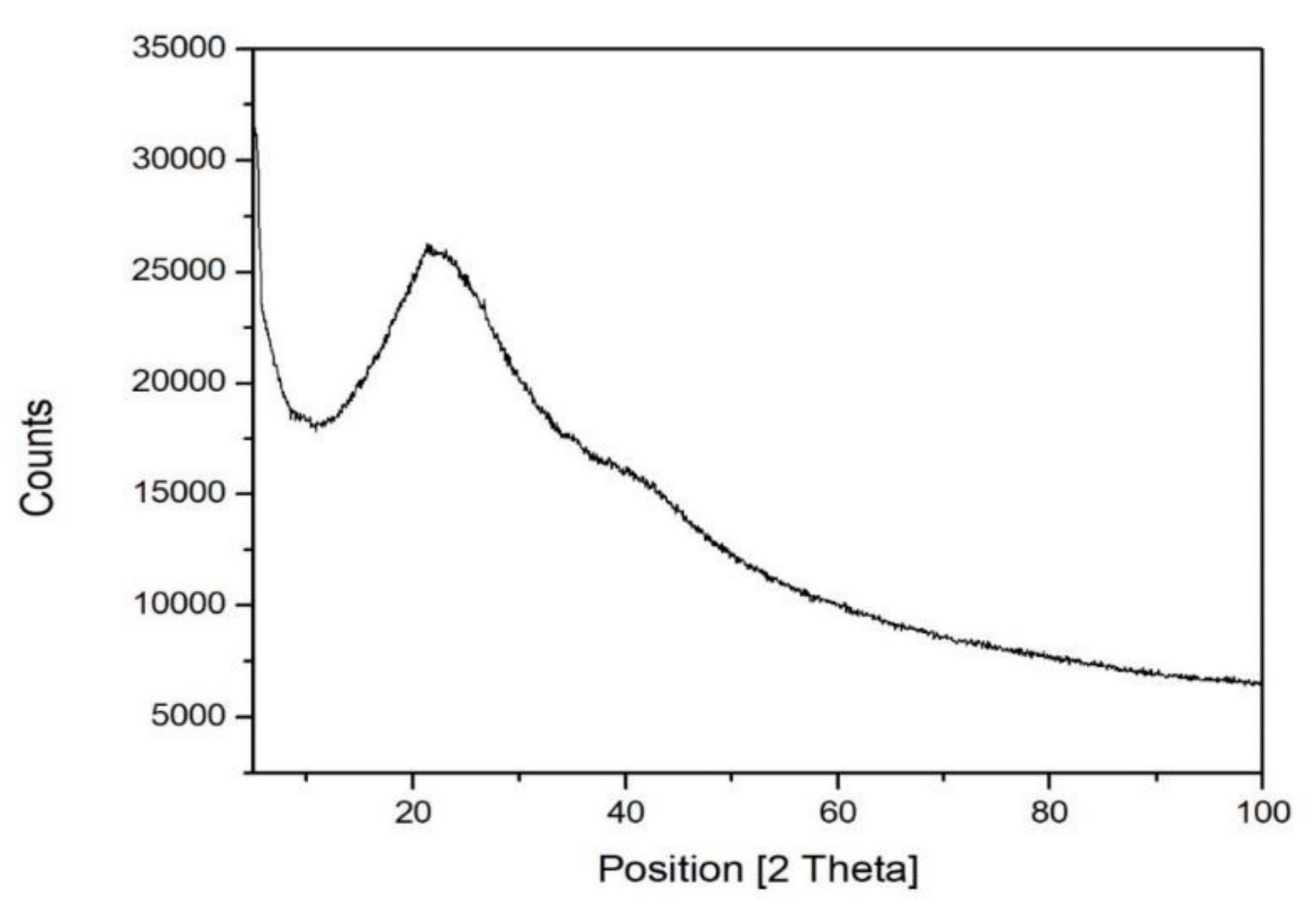
X-ray diffraction (XRD) spectrum of the synthesized copper nanoparticles.

X-ray diffraction analysis confirms the amorphous nature of the synthesized copper nanoparticles (Figure 5). This substantiates the electron diffraction pattern observed in the HRTEM images (Figure 2), suggesting that the synthesized copper nanoparticles are amorphous. Since the nanomaterial was synthesized using a plant leaf extract, the agglomeration occurs due to the encapsulation of nanomaterial by the phytochemicals. These organic moieties form layers of sheets of agglomeration. Using X-ray diffraction (XRD) analysis, we can observe only long-range disorder in the spectrum.

#### 2.2.2. Fourier-Transform Infrared (FTIR) Spectroscopy of the M. oleifera Leaves Extract and the Synthesized Copper Nanoparticles

**Figure 6:**
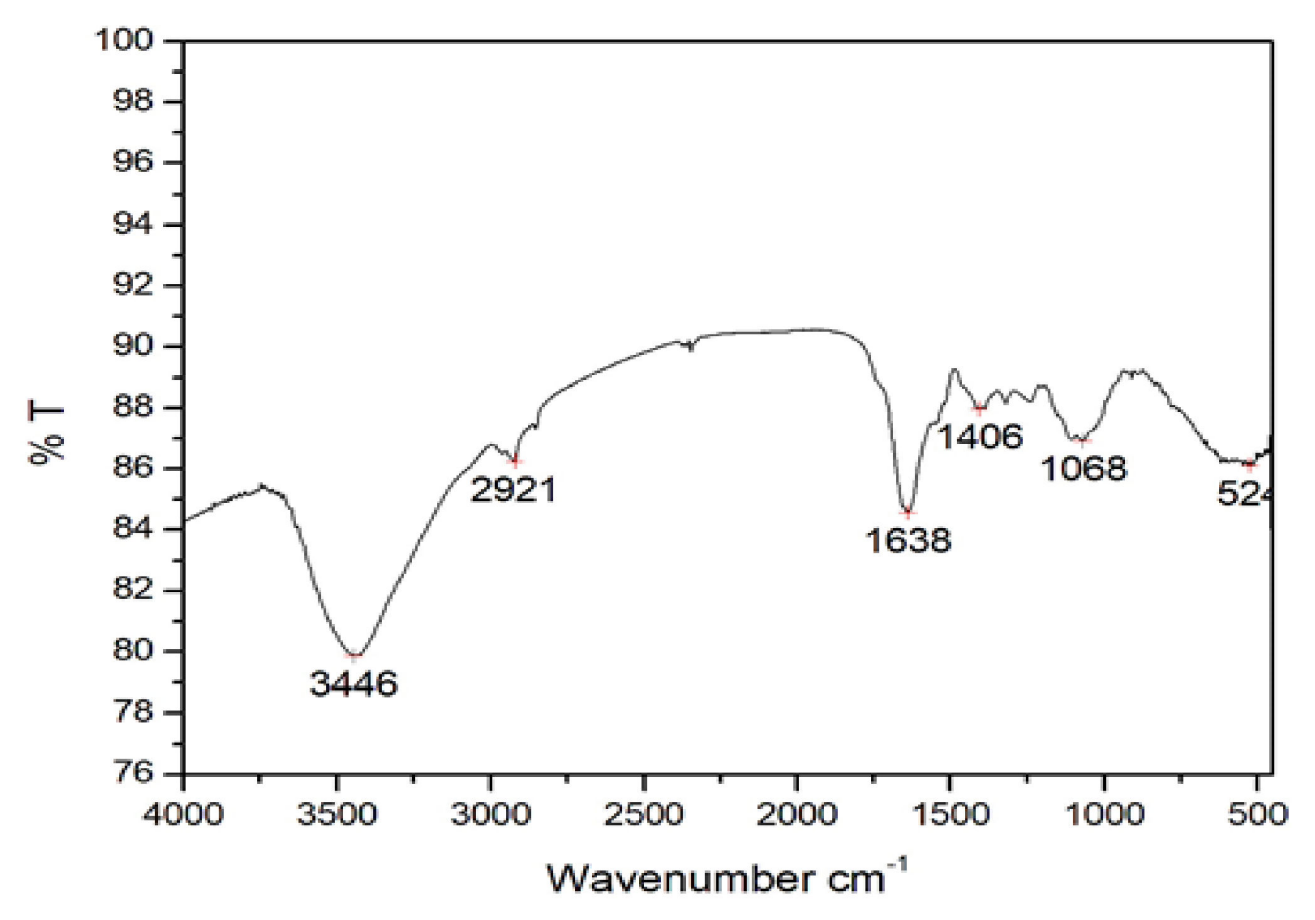
Fourier-Transform Infrared (FTIR) spectrum of the *M. oleifera* leaves extract.

**Figure 7:**
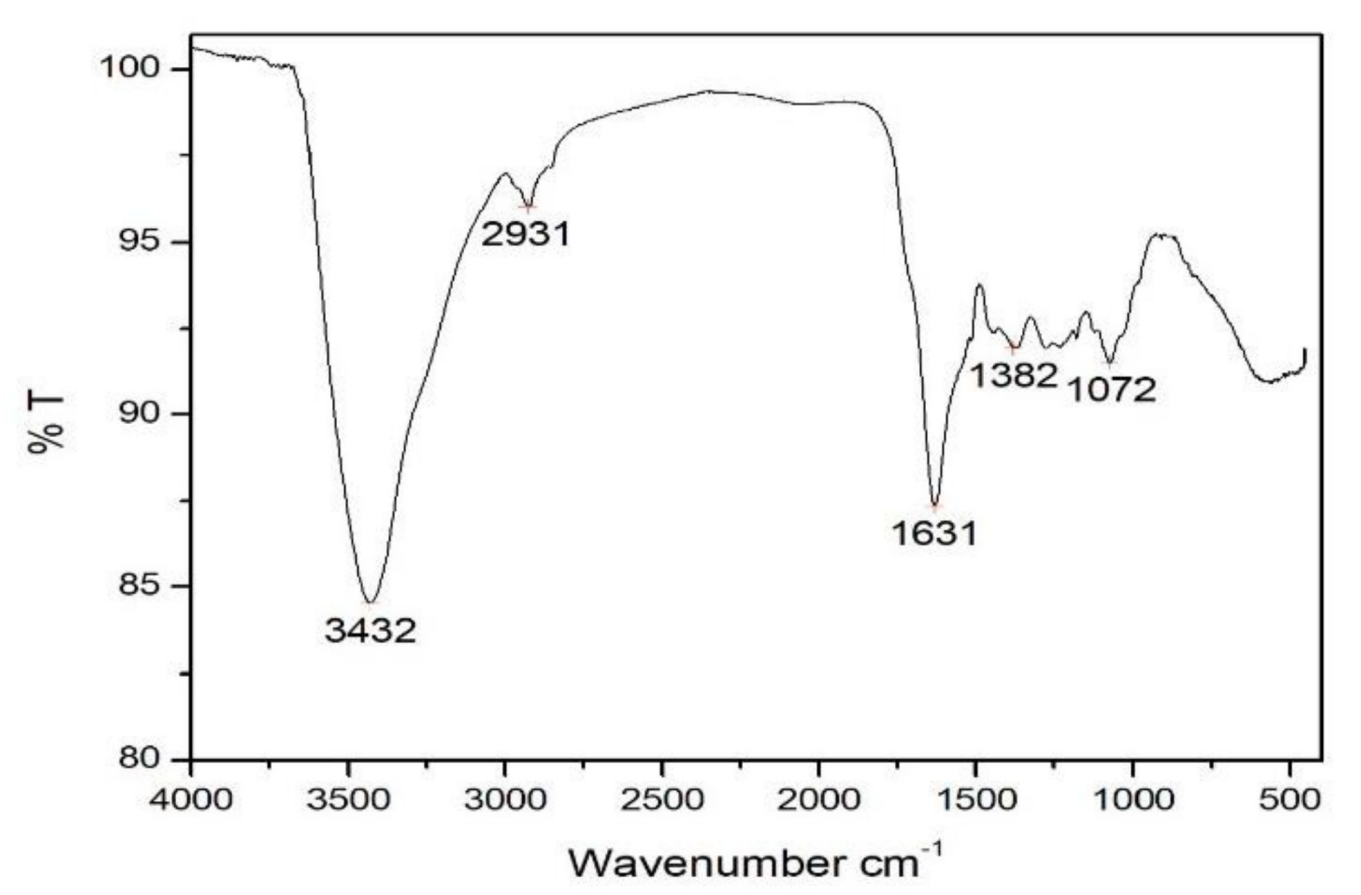
FTIR spectrum of the synthesized copper nanoparticles.

The FTIR spectra of the *M. oleifera* leaves extract and the synthesized copper nanoparticles are shown in Figure 6 and Figure 7, respectively. This vibration spectroscopy data can be used to understand the biomolecules involved in the synthesis of copper nanoparticles. The bands around 3400 cm^−1^ and 1630 cm^−1^ are broad in the *M. oleifera* leaves extract (Figure 6), corresponding to the vibration mode of a hydroxyl group, mostly found in polyphenolic molecules such as tannins, flavonoids, and glycoside derivatives. The signals at 1638 cm^−1^ and 1068 cm^−1^ correspond to N-H bending and C-N bond stretching, respectively.

The FTIR spectrum of the synthesized copper nanoparticles (Figure 7) depicts a sharp narrow band at 3432 cm^−1^, corresponding to the N-H vibration mode. The FTIR of the extract gave a broad signal at 3400 cm^−1^, corresponding to hydrogen-bonded poly-hydroxyl groups of the phytochemicals present in encapsulated copper nanoparticles. The signals at 1631 cm^−1^ and 1072 cm^−1^ correspond to N-H bending and C-N bond stretching respectively.

This comparison shows the participation of the hydroxyl group in the synthesis of copper nanoparticles. Moreover, the binding characteristics of amino groups in the *M. oleifera* leaves extract and their signals in the FTIR spectrum of the synthesized copper nanoparticles confirm the encapsulation of copper nanomaterial during the synthesis (Figure 6 and Figure 7). This could be the reason for agglomeration of the synthesized copper nanoparticles to give an amorphous morphology.

#### 2.2.3. Ultraviolet-Visible Light (UV-Vis) Spectroscopy of the M. oleifera Leaves Extract and the Synthesized Copper Nanoparticles

**Figure 8:**
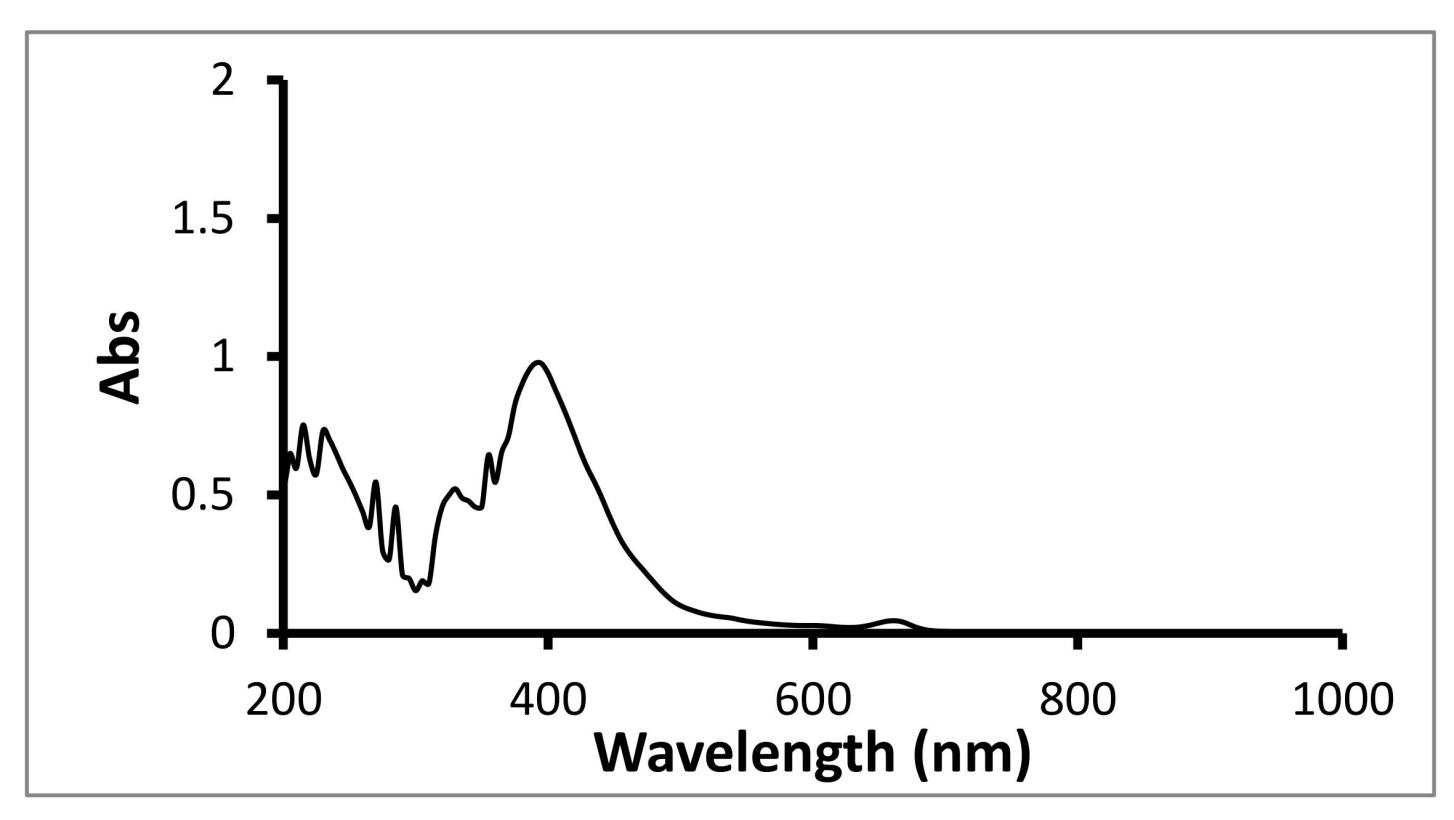
UV-Vis spectrum of the *M. oleifera* leaves extract.

**Figure 9:**
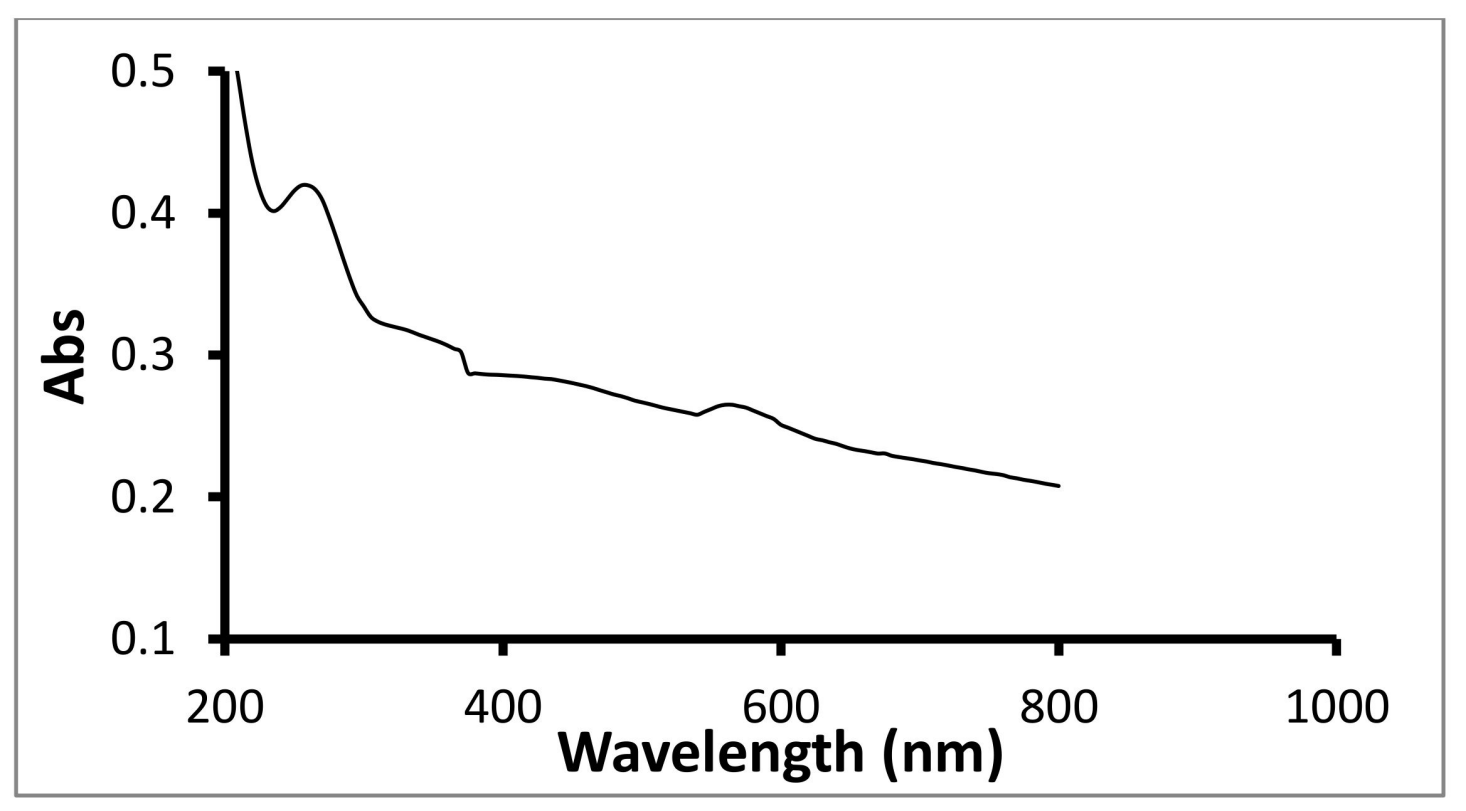
UV-Vis spectrum of the synthesized copper nanoparticles.

The UV-Vis absorption spectrum of the *M. oleifera* leaves extract is shown in Figure 8, with λ_max_ at 390 nm. The UV-Vis spectrum of the synthesized copper nanoparticles reconstituted in dimethyl sulfoxide (DMSO) solvent is shown in Figure 9, with λ_max_ at 260 nm.

The copper nanoparticles usually absorb from 280 nm to 360 nm, while absorbance is usually detected at 620 nm for the aqueous copper sulfate solution. The detection of λ_max_ at 260 nm and absence of absorbance at 620 nm confirms the reduction of copper ions in the aqueous solution and formation of copper nanoparticles.

### 2.3. Antioxidant Activity of the M. oleifera Leaves Extract and the Synthesized Copper Nanoparticles

Using DPPH assay against ascorbic acid as a standard, the percentage of antioxidant activity of the *M. oleifera* leaves extract and the synthesized copper nanoparticles was assessed. As shown in Table 3, the *M. oleifera* leaves extract exerted considerable antioxidant activity, while the synthesized copper nanoparticles displayed a lower activity. These results were supported with total antioxidant capacity measured by phosphomolybdate assay (Table 4).

### 2.4. Anti-Bacterial Activity of the M. oleifera Leaves Extract and the Synthesized Copper Nanoparticles

The potential anti-bacterial activity of the synthesized copper nanoparticles was evaluated against *Escherichia coli*, *Klebsiella pneumoniae*, *Staphylococcus aureus*, and *Enterococcus faecalis*. Streptomycin (10 µg/500 µL) was used as a positive control, while water-ethanol (1:1) solution and DMSO solvent were used as negative controls for the *M. oleifera* leaves extract and the copper nanoparticles, respectively. The nutrient broth was also used as a negative control. The minimum inhibitory concentration (MIC) values measured in presence of the *M. oleifera* leaves extract and the copper nanoparticles are given in Table 5. The growth of the indicated species of bacteria in presence of different concentrations (7.8–1000 µg/mL) of *M. oleifera* leaves extract and the copper nanoparticles is shown in Table 6. In presence of the *M. oleifera* leaves extract, the MIC values against *Escherichia coli*, *Klebsiella pneumoniae*, *Staphylococcus aureus*, and *Enterococcus faecalis* were in the range of 250–500 µg/mL (Table 5). The anti-bacterial activity of the synthesized copper nanoparticles was observed with MIC values (250–500 µg/mL) (Table 5). The anti-bacterial activity (MIC values) of the synthesized copper nanoparticles was comparable to that observed for the *M. oleifera* leaves extract (Table 6). A sample of resazurin microtiter assay plates for the *M. oleifera* leaves extract and the synthesized copper nanoparticles is given in Figure S1.

### 2.5. Anti-Fungal Activity of the M. oleifera Leaves Extract and the Synthesized Copper Nanoparticles

The potential anti-fungal activity of synthesized copper nanoparticles was evaluated against *Aspergillus niger*, *Aspergillus flavus*, *Candida albicans*, and *Candida glabrata*. Ketoconazole (10 µg/500 µL) was used as a positive control, while water-ethanol (1:1) solution and DMSO solvent were used as negative controls for the *M. oleifera* leaves extract and the copper nanoparticles, respectively. The nutrient broth was also used as a negative control. The minimum inhibitory concentration (MIC) values measured for the *M. oleifera* leaves extract and the copper nanoparticles are given in Table 7. The growth of the indicated species of fungi in presence of different concentrations (7.8–1000 µg/mL) of *M. oleifera* leaves extract and the copper nanoparticles is shown in Table 8. The copper nanoparticles displayed more effective anti-fungal activity against *Candida albicans* and *Candida glabrata* than the *M. oleifera* leaves extract (Table 7 and Table 8). In presence of the *M. oleifera* leaves extract, the MIC values against *Aspergillus niger*, *Aspergillus flavus*, *Candida albicans*, and *Candida glabrata* were found to be 62.5, 62.5, 125, and 250 µg/mL, respectively (Table 7). In presence of the synthesized copper nanoparticles, the MIC values against *Aspergillus niger*, *Aspergillus flavus*, *Candida albicans*, and *Candida glabrata* were found to be 125, 125, 62.5, and 31.2 µg/mL, respectively (Table 7). A sample of resazurin microtiter assay plates for the *M. oleifera* leaves extract and the synthesized copper nanoparticles is given in Figure S2. These findings indicate that the copper nanoparticles displayed more effective anti-fungal activity against *Candida albicans* and *Candida glabrata* compared to the *M. oleifera* leaves extract (Table 7 and Table 8). Moreover, it is concluded that the enhanced anti-fungal activity of copper nanoparticles against *Candida* species makes them a better therapeutic choice for direct application as antimicrobial agents.

## 3. Materials and Methods

### 3.1. Preparation of the M. oleifera Leaves Extract

The leaves of *M. oleifera* were collected from the wild shrub regions in Rajapalayam, Tamil Nadu, India. The plant was identified by the facility at Asthagiri Herbal Research Foundation, Chennai, India. The identified specimen vouchers (AHRF/HERBARIUM/021) were deposited at Asthagiri Herbal Research Foundation, Chennai, India. The country’s common breed was used in the study. The genetically-modified varieties of *M. oleifera* (PKM-1 and PKM-2) were not used in our study. Fresh *M. oleifera* leaves were collected and shade-dried. The dried leaves were smoothly crushed. The *M. oleifera* leaves powder was stored at room temperature. A total of 10 g of *M. oleifera* leaves powder was taken and soaked in 100 mL water-ethanol (1:1) solution. The mixture was macerated for 1 h and the *M. oleifera* leaves extract was filtered through Whatman filter paper 1. The *M. oleifera* leaves extract was used immediately for the preparation of copper nanoparticles and other experimental analyses.

### 3.2. Synthesis of the Copper Nanoparticles

Copper sulfate pentahydrate (1g) was dissolved in 20 mL de-mineralized water and added to 80 mL of *M. oleifera* leaves extract. The reaction mixture was stirred for 3 h at 60 °C. The synthesized copper nanoparticles were collected by centrifugation and repeatedly washed with de-mineralized water. The synthesized copper nanoparticles were dried at 105 °C for 1 h, and subsequently reconstituted in DMSO solvent.

### 3.3. Characterization of the Size and Morphology of the Synthesized Copper Nanoparticles

The completion of synthesis was characterized using a UV-3600 Plus UV-Vis double beam spectrophotometer (Shimadzu, Kyoto, Japan). The formation of copper nanoparticles and the role of biomolecules in this synthesis were confirmed using an ALPHA-E FTIR spectrometer (Bruker, Billerica, MA, USA). The crystalline nature of the synthesized copper nanoparticles was ascertained by XRD using a XRD-6000 diffractometer (Shimadzu, Kyoto, Japan). The size of the synthesized copper nanoparticles was measured by SEM using a FEI Quanta 200 scanning electron microscope, with the field emission gun (FEG) feature for better resolution (ThermoFisher Scientific, Waltham, MA, USA). The morphology assessment of the synthesized nanoparticles was performed by transmission electron microscopy (TEM) using a JEOL-TEM-2100 plus transmission electron microscopy (JEOL, Tokyo, Japan). The sample was dispersed in ethanol, coated on the grid, and dried for TEM analysis along with EDS analysis.

### 3.4. Phytochemical Analysis of the M. oleifera Leaves Extract

The qualitative analysis of biomolecules present in the *M. oleifera* leaves extract was carried out for the presence of alkaloids, tannins, flavonoids, steroids, saponins, polyphenols, glycosides, carbohydrates, proteins, and amino acids. Experimental details of the phytochemical analysis are given in Supplementary Materials. The total phenolic content in the *M. oleifera* leaves extract was estimated as gallic acid equivalent by Folin–Ciocalteu polyphenol assay [36]. The protein content in the *M. oleifera* leaves extract was estimated by Lowry’s method [37].

### 3.5. Antioxidant Activity

#### 3.5.1. DPPH Assay (Antioxidant Activity Percentage—AA%)

The antioxidant activity percentage (AA%) (scavenging activity) of the *M. oleifera* leaves extract and the synthesized copper nanoparticles was assessed by DPPH free radical scavenging assay. A total of 1 mg of ascorbic acid (standard) was dissolved in 1 mL methanol. Different aliquots (serial dilution) of the ascorbic acid solution (0.1–0.5 mL), corresponding to 100–500 µg, were used for calibration. To each tube containing ascorbic acid solution, 1 mL of 0.1 mM DPPH radical solution in ethanol was added, and the final volume was adjusted to 4 mL using ethanol. The stock solutions for the *M. oleifera* leaves extract and the synthesized copper nanoparticles were prepared by dissolving 1 mg of each sample in 1 mL of an appropriate solvent (methanol for the *M. oleifera* leaves extract; DMSO for the synthesized copper nanoparticles). Different aliquots from the stock solutions (0.1–0.5 mL), corresponding to 100–500 µg, were added to all tubes except the blank tube (control). The volume in each tube was adjusted to 3 mL using ethanol. To each tube, 1 mL of 0.1 mM DPPH radical solution in ethanol was added. The blank tube (control) was prepared by mixing 3 mL of ethanol and 1 mL of DPPH radical solution in ethanol. All tubes were incubated for 30 min at room temperature, and absorbance at 517 nm was recorded. AA% was determined using the following formula:

AA% = [(absorbance of blank) − (absorbance of sample)/(absorbance of blank)] × 100

#### 3.5.2. Phosphomolybdenum Assay (Total Antioxidant Capacity—TAC)

The total antioxidant capacity (TAC) of the *M. oleifera* leaves extract and the synthesized copper nanoparticles was assessed by phosphomolybdenum assay as ascorbic acid equivalent. A total of 1 mg of ascorbic acid (standard) was dissolved in 1 mL methanol. Different aliquots (serial dilution) of the ascorbic acid solution (0.1–0.5 mL), corresponding to 100–500 µg, were prepared. The volume in each tube was adjusted to 4 mL using distilled water. The stock solutions for the *M. oleifera* leaves extract and the synthesized copper nanoparticles were prepared by dissolving 1 mg of each sample in 1 mL of an appropriate solvent (methanol for the *M. oleifera* leaves extract; DMSO for the synthesized copper nanoparticles). Different aliquots from the stock solutions (0.1–0.5 mL), corresponding to 100–500 µg, were added to all tubes except the blank tube (control). The volume in each tube was adjusted to 3 mL using distilled water. To each tube, 1 mL of phosphomolybdenum reagent (0.6 M sulphuric acid, 28 mM sodium phosphate, and 4 mM ammonium molybdate) was added. The volume in each tube was adjusted to 4 mL using distilled water. After incubation for 90 min at 95 °C, absorbance at 695 nm was recorded. The calibration curve for the ascorbic acid solution (standard) was plotted for the absorbance at 695 nm against known amounts of ascorbic acid with the phosphomolybdate reagent, so as to express the TAC values as ppm equivalent of ascorbic acid. Using the ascorbic acid calibration curve, the TAC values for the *M. oleifera* leaves extract and the synthesized copper nanoparticles were calculated and expressed as ppm equivalent of ascorbic acid.

### 3.6. Anti-Bacterial Activity using Resazurin Microtiter Assay

The most rapid and inexpensive way to screen several microorganism isolates at the same time, with better correlation in comparison to other techniques, is the resazurin microtiter assay [38,39,40]. The resazurin solution was prepared by dissolving a 270 mg tablet of resazurin in 40 mL of sterile distilled water. The test was carried out in 96-well plates under aseptic conditions. As a standard protocol, a loop full of bacterial culture was inoculated in 10 mL of the appropriate broth medium for 6 h at 37 °C in a Bio-Oxygen Demand (BOD) incubator. A volume of 100 μL of sample containing 10 mg/mL was pipetted into the first well of the plate. To all other wells, 50 μL of nutrient broth was added, and the tested sample was serially diluted. Subsequently, 10 μL of resazurin solution and 10 μL of bacterial suspension were added to each well. Each plate was wrapped loosely with cling film to prevent dehydration. The plates were incubated at 37 °C for 18–24 h. The color change was then assessed visually. A color change from purple to pink (or colorless) was recorded as positive, indicating cell growth (i.e., (+) means growth and (-) means no growth). The lowest concentration at which color change occurred was considered to be MIC. Streptomycin (10 µg/500 µL) was used as a positive control, while water-ethanol (1:1) solution and DMSO solvent were used as negative controls for the *M. oleifera* leaves extract and the copper nanoparticles, respectively. The nutrient broth was also used as a negative control.

### 3.7. Anti-Fungal Activity Using Resazurin Microtiter Assay

The resazurin solution was prepared by dissolving a 270 mg tablet of resazurin in 40 mL of sterile distilled water. The test was carried out in 96-well plates under aseptic conditions. As a standard protocol, a loop full of fungal culture was inoculated in 10 mL of the appropriate broth medium for 6 h at 37 °C in a Bio-Oxygen Demand (BOD) incubator. A volume of 100 μL of sample containing 10 mg/mL was pipetted into the first well of the plate. To all other wells, 50 μL of nutrient broth was added, and the tested sample was serially diluted. Subsequently, 10 μL of resazurin solution and 10 μL of fungal suspension were added to each well. Each plate was wrapped loosely with cling film to prevent dehydration. The plates were incubated at 37 °C for 18–24 h. The color change was then assessed visually. A color change from purple to pink (or colorless) was recorded as positive, indicating cell growth (i.e., (+) means growth and (-) means no growth). The lowest concentration at which color change occurred was considered to be MIC. Ketoconazole (10 µg/500 µL) was used as a positive control, while water-ethanol (1:1) solution and DMSO solvent were used as negative controls for the *M. oleifera* leaves extract and the copper nanoparticles, respectively. The nutrient broth was also used as a negative control.

## 4. Conclusions

The green synthesis of copper nanoparticles using a hydroalcoholic extract of *M. oleifera* leaves was successful. The formation of copper nanoparticles and the role of biomolecules in this synthesis were confirmed by UV-Vis absorption. FTIR spectrometry supports the encapsulation of copper nanoparticles by the phytochemicals present in the *M. oleifera* leaves. The amorphous nature of the synthesized copper nanoparticles encapsulated by phytochemicals was ascertained by XRD and the electron diffraction pattern from HRTEM images. The particle size of the synthesized copper nanoparticles was measured to be 35.8–49.2 nm using SEM imaging. EDS analysis confirms the presence of copper element in synthesized encapsulated nanoparticles. Our study reveals that the *M. oleifera* leaves extract and the synthesized copper nanoparticles display considerable antioxidant activity. We also demonstrate that the *M. oleifera* leaves extract and the synthesized copper nanoparticles exert considerable anti-bacterial activity against *Escherichia coli*, *Klebsiella pneumoniae*, *Staphylococcus aureus*, and *Enterococcus faecalis*, with MIC values in the range of 250–500 µg/mL. Similarly, the *M. oleifera* leaves extract and the synthesized copper nanoparticles exert relatively stronger anti-fungal activity against *Aspergillus niger*, *Aspergillus flavus*, *Candida albicans*, and *Candida glabrata*, with MIC values in the range of 62.5–250 µg/mL and 31.2–125 µg/mL, respectively. Indeed, the anti-fungal activity of the synthesized copper nanoparticles is more effective than that of the *M. oleifera* leaves extract against *Candida albicans* and *Candida glabrata*. We also conclude that the anti-bacterial and anti-fungal activities were not deterred in the process of green synthesis of the copper nanoparticles. These findings suggest that the synthesized copper nanoparticles can be a promising therapeutic candidate for the treatment of various bacterial, and particularly, fungal infections such as candidiasis. Due to encapsulation, the synthesized copper nanoparticles can be employed in direct medical application as antimicrobial agents. Yielding non-toxic material and reducing the production of wasteful products is a huge advantage of green synthesis of nanoparticles. Moreover, compared to the incinerated nanoparticles that are synthesized by conventional thermal-chemical methods, the described green synthesis approach leading to the synthesis of nanoparticles encapsulated with phytochemicals/polyphenols is of significant therapeutic value.

## Figures and Tables

**Table 1:** Phytochemical analysis of the *Moringa oleifera* leaves extract. The presence is indicated with (+).

Functional Group	Test Method	*M. oleifera* Leaves Extract
Alkaloids	Dragendroff’s test	+
Tannins	Ferric chloride test	+
Flavonoids	Shinoda test	+
Steroids	Salkowski reaction test	+
Saponins	Foam test	+
Polyphenols	Puncal-D test	+
Glycosides	Conc. H_2_SO_4_ and heat	+
Carbohydrates	Anthrone test	+
Proteins	Ninhydrin test	+
Amino acids	Millon’s test	+

**Table 2:** Estimation of total phenolic content before and after the synthesis of copper nanoparticles.

Sample	Total Phenolic Content (mg/g of Dried Leaves)
*M. oleifera* leaves extract (before synthesis)	23.0 ± 0.3
*M. oleifera* leaves extract (after synthesis)	17.0 ± 0.4

**Table 3:** Antioxidant activity percentage (AA%) using DPPH assay.

Sample	Amount (µg)				
	100	200	300	400	500
Ascorbic acid (standard)	34.4	55.1	67.2	75.8	84.4
*M. oleifera* leaves extract	55.1	58.6	63.7	65.5	65.5
Copper nanoparticles	12.0	13.7	17.2	20.6	29.3

**Table 4:** Total antioxidant capacity (TAC) using phosphomolybdate assay.

Sample	Concentration (µg/mL) Ascorbic Acid Equivalent				
	50	100	150	200	250
*M. oleifera* leaves extract	32.5	65.0	102.5	132.5	172.5
Copper nanoparticles	12.5	12.5	17.5	25.0	47.5

**Table 5:** Anti-bacterial activity (MIC values).

Bacterial Species	MIC (µg/mL)
*M. oleifera* leaves extract	
*Escherichia coli*	500
*Klebsiella pneumoniae*	250
*Staphylococcus aureus*	250
*Enterococcus faecalis*	250
Copper nanoparticles	
*Escherichia coli*	500
*Klebsiella pneumoniae*	500
*Staphylococcus aureus*	500
*Enterococcus faecalis*	250

**Table 6:** Anti-bacterial activity data (growth (+) and no growth (-)).

No.	Bacterial Species	Growth of Bacteria										
	Concentration (µg/mL)	1000 (1)	500 (2)	250 (3)	125 (4)	62.5 (5)	31.2 (6)	15.6 (7)	7.8 (8)	Streptomycin (10 µg/500 µL)	Negative Control	Nutrient Broth
	*M. oleifera* leaves extract											
1	*Escherichia coli*	-	-	+	+	+	+	+	+	-	+	+
2	*K. pneumoniae*	-	-	-	+	+	+	+	+	-	+	+
3	*S. aureus*	-	-	-	+	+	+	+	+	-	+	+
4	*E. faecalis*	-	-	-	+	+	+	+	+	-	+	+
	Copper Nanoparticles											
1	*E. coli*	-	-	+	+	+	+	+	+	-	+	+
2	*K. pneumoniae*	-	-	+	+	+	+	+	+	-	+	+
3	*S. aureus*	-	-	+	+	+	+	+	+	-	+	+
4	*E. faecalis*	-	-	-	+	+	+	+	+	-	+	+

**Table 7:** Anti-fungal activity (MIC values).

Fungal Species	MIC (µg/mL)
*M. oleifera* leaves extract	
*Aspergillus niger*	62.5
*Aspergillus flavus*	62.5
*Candida albicans*	125
*Candida glabrata*	250
Copper nanoparticles	
*Aspergillus niger*	125
*Aspergillus flavus*	125
*Candida albicans*	62.5
*Candida glabrata*	31.2

**Table 8:** Anti-fungal activity data (growth (+) and no growth (-)).

No.	Fungal Species	Growth of Fungi										
	Concentration (µg/mL)	1000 (1)	500 (2)	250 (3)	125 (4)	62.5 (5)	31.2 (6)	15.6 (7)	7.8 (8)	Ketoconazole (10 µg/500 µL)	Negative Control	Nutrient Broth
*M. oleifera* leaves extract												
1	*A. niger*	-	-	-	-	-	+	+	+	-	+	+
2	*A. flavus*	-	-	-	-	-	+	+	+	-	+	+
3	*C. albicans*	-	-	-	-	+	+	+	+	-	+	+
4	*C. glabrata*	-	-	-	+	+	+	+	+	-	+	+
Copper Nanoparticles												
1	*A. niger*	-	-	-	-	+	+	+	+	-	+	+
2	*A. flavus*	-	-	-	-	+	+	+	+	-	+	+
3	*Ca. albicans*	-	-	-	-	-	+	+	+	-	+	+
4	*C. glabrata*	-	-	-	-	-	-	+	+	-	+	+

## Supplementary Materials

The following are available online, Figure S1: Resazurin microtiter assay plates for the *M. oleifera* leaves extract and the synthesized copper nanoparticles, Figure S2: Resazurin microtiter assay plates for the *M. oleifera* leaves extract and the synthesized copper nanoparticles, Experimental details: Phytochemical tests.
