# Supplementary material for: Green Synthesis of Encapsulated Copper Nanoparticles Using a Hydroalcoholic Extract of Moringa oleifera Leaves and Assessment of Their Antioxidant and Antimicrobial Activities
Source: Molecules. 2020 Jan 28;25(3):555. doi: 10.3390/molecules25030555 (PMC7037650; doi:10.3390/molecules25030555)
Supplement: Supplementary file 1 [file molecules-25-00555-s001.pdf]

Supplementary Material

Green synthesis of encapsulated copper nanoparticles using a hydroalcoholic extract of *Moringa oleifera* leaves and assessment of their antioxidant and anti-microbial activities

Prince Edwin Das,[1] Imad A. Abu-Yousef,[2]* Amin F. Majdalawieh,[2] Srinivasan Narasimhan,[1]* and Palmiro Poltronieri[3]*

[1]Asthagiri Herbal Research Foundation, 162A, Perungudi Industrial Estate, Perungudi, Chennai, India 600096. E-mails: prince.ahrf@gmail.com (P.E.D.); asthagiri.herbal@gmail.com (S.N.)

[2]Department of Biology, Chemistry and Environmental Sciences, American University of Sharjah, P.O. Box 26666, Sharjah, United Arab Emirates. E-mails: iabuyousef@aus.edu (I.A.A.Y); amajdalawieh@aus.edu (A.F.M)

[3]Institute of Sciences of Food Productions, CNR-ISPA, Lecce 73100, Italy. E-mail: palmiro.poltronieri@ispa.cnr.it (P.P.)

**Corresponding Authors.* I.A. Abu-Yousef: Phone: (971) 6 5152410; Fax: (971) 6 515 2450. E-mail: iabuyousef@aus.edu; *S.* Narasimhan: Phone: (91) 44 22397645; Fax: (91) 44 22397645. E-mail: narasimhan_s@yahoo.com; asthagiri.herbal@gmail.com; P. Poltronieri: palmiro.poltronieri@ispa.cnr.it; Tel.: (39) 83 2422609; Fax: (39) 83 2422620

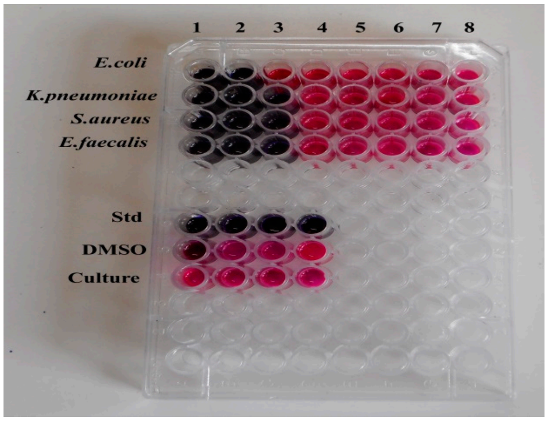

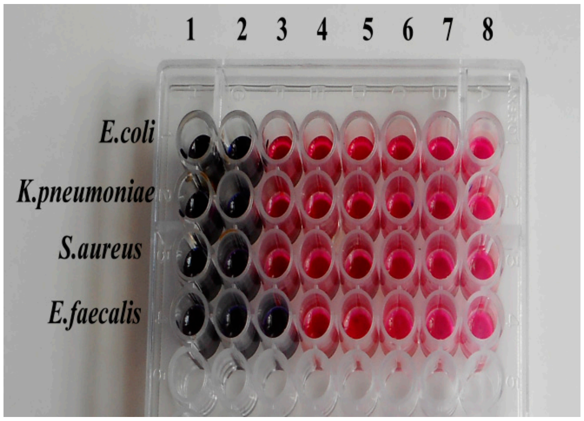

M. oleifera leaves extract

Copper nanoparticles

Supplementary Figure 1. Resazurin microtiter assay plates for the *M. oleifera* leaves extract and the synthesized copper nanoparticles.

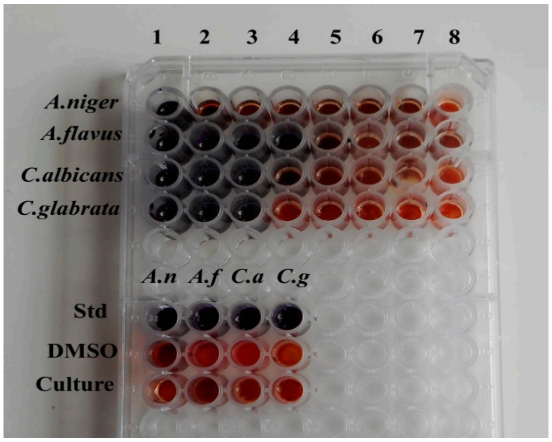

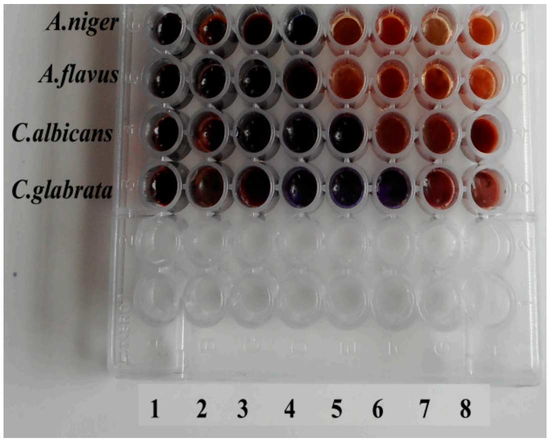

M. oleifera leaves extract

Copper nanoparticles

Supplementary Figure 2. Resazurin microtiter assay plates for the *M. oleifera* leaves extract and the synthesized copper nanoparticles.

Supplementary Document 1

A brief description of the tests used for the phytochemical analysis. All tests are described in [33]. These are standard, commonly used tests. The experimental procedures are shown below.

Alkaloids
Dragendorff's test:
To 2 mL of the extract added 1 mL of Dragendorff's reagent along the side of the test tube. Formation of orange or orange reddish brown precipitate indicated the presence of alkaloids.

Tannins
Ferric chloride test:
This detection was based on blue colour formed by the addition of few drops of 5% ferric chloride solution to 2 mL of the extract solution.

Flavonoids
Shinoda test:
A few magnesium turnings and 5 drops of concentrated hydrochloric acid was added drop wise to 1 mL of the extract solution. A pink, scarlet, crimson red or occasionally green to blue colour appeared after few minutes confirm the presence of flavonoids.

Steroids
Salkowski reaction test:
2 mg of dried extract was shaken with 1 mL of chloroform and a few drops of concentrated sulphuric acid were added along the side of the test tube. A red brown colour formed at the interface of two layers indicates the presence of steroids.

Saponins
Foam test:
5 mL of the extract was taken in a test tube was shacked well for five minutes. Formation of stable foam indicates the presence of saponins.

Polyphenols
Puncal-D test:
2 mL of the extract solution added to 1 mL of puncal-D reagent in a test tube. The fluorescent blue colour indicates the presence of polyphenols.

Glycosides
Conc. H_2SO_4 and heat:
2 mL of the extract solution was added to 1 mL concentrated sulphuric acid and the mixture was warmed. The appearance of charred black colour indicates the presence of glycosides.

Carbohydrates
Anthrone test:
2 mL of the extract solution, 1 mL of Anthrone reagent was added and the solution was warmed. The solution was set aside to give a bluish green colour, indicates the presence of carbohydrates.

Proteins

Ninhydrin test:

2 mL of extract solution was added to 1 mL of Ninhydrin reagent in alcohol. The formation of blue or deep blue colour indicates the presence of proteins.

Amino acids

Millon's test:

5 drops of Millon's reagent were added to 2 mL of extract solution and heated on water bath for 10 min, cooled and added 1% sodium nitrite solution. Appearance of red colour confirms the presence of amino acids.
